# Impact of Lockdown due to COVID-19 on the Modalities of Intoxicated Patients Presenting to the Emergency Room

**DOI:** 10.1017/S1049023X20001533

**Published:** 2021-01-05

**Authors:** Manar M. Fayed, Asmaa F. Sharif

**Affiliations:** 1. Forensic Medicine and Clinical Toxicology Department, Faculty of Medicine, Tanta University, Egypt; 2. Clinical Medical Sciences Department, College of Medicine, Dar Al Uloom University, Saudi Arabia

**Keywords:** antipsychotics, COVID-19, drug poisoning, lockdown, pesticides

## Abstract

**Introduction:**

Coronavirus disease 2019 (COVID-19) pandemic influences health care facilities world-wide. The flow rate, type, and severity of cases presented to emergency departments varied during the pandemic in comparison to the past years. However, this change has not been well-described among the cases of hospital admission due to toxic exposure.

**Study Objective:**

Recognition of the pattern of toxic exposure among the cases refereed to Tanta Poison Control Center (TPCC; Tanta, Egypt) during the past five years, and furthermore, exploration of the impact of lockdown due to the COVID-19 pandemic on the pattern of presented cases.

**Methods:**

The current study is a five-year retrospective, comparative cross-sectional study carried out among acutely intoxicated patients admitted to TPCC during the spring months (March through May) of 2016-2020. A total of 1,916 patients with complete medical records were recruited. The type and manner of toxic exposure, demographic, clinical data, and outcomes were analyzed.

**Results:**

The current study noted that there were delays in time from toxic exposure to emergency services during the lockdown period. This was reflected in significant lower recovery rates (884.8/1,000 population; z = −3.0) and higher death rates (49.4/1,000 population; z = 2.1) despite the marked decrease in the total number of hospital admissions in comparison to the past four years. The lockdown period showed significantly higher phosphides (z = 3.5; χ^2^ = 34.295; P <.001) and antipsychotics exposure (z = 3.6; χ^2^ = 21.494; P <.001) than the previous years. However, predominance of female exposure and intentional self-poisoning was maintained over the past five years, including the lockdown.

**Conclusion:**

COVID-19-associated lockdown greatly reformed the usual intoxication pattern of the cases admitted to emergency room. Also, it played a role in delaying time of hospital arrival, which was reflected as lower recovery rates and higher death rates.

## Introduction

Coronavirus disease 2019 (COVID-19) is the disease caused by severe acute respiratory syndrome-coronavirus-2 (SARS-CoV-2). It was first identified in Wuhan, China in December 2019.^1,2^ By February 14, 2020, the first case appeared in Egypt. The World Health Organization (WHO; Geneva, Switzerland) declared a pandemic on March 11, 2020.^3^

There is no proven vaccination against SARS-CoV-2. Strong infection control measures and public adherence are the primary interventions to minimize viral spread.^4–6^

General public measures were suggested, including telework, closure of non-essential facilities, and even complete lockdown.^7^ The COVID-19 pandemic poses a unique challenge to health care delivery. Telemedicine is not feasible in emergencies such as acute poisoning.^8,9^

In Egypt, a partial lockdown was implemented on March 12, resulting in serious impacts on the economy and mental health.^10^ Lockdowns in the United Kingdom led to difficulty in accessing treatment and emergency departments quickly.^11^ Italy showed reductions of as much as 73% to 88% in pediatric emergency department visits in comparison to 2019 and 2018.^12^

The WHO reported three million annual hospital admissions due to toxic exposure world-wide, and 99% of poisonings that occur in developing countries are lethal.^13^ Developing countries report high mortality due to increasing rates of toxic exposure, both intentional and unintentional.^14^

Recognition of the pattern of poisonings in a given area may help to predict the need for prophylaxis, early diagnosis, and proper health care utilization to reduce morbidity and mortality during the pandemic.

Since patients with acute poisoning usually present as emergency cases, delayed transfer or improper management may lead to serious deterioration. The current study aims to study the pattern of cases of acute poisoning admitted to the Tanta Poison Control Center (TPCC; Tanta, Egypt) in the spring (March through May) of 2016 through 2020 and to assess the impact of COVID-19 lockdown on this pattern.

## Methods

### Study Design

The current study is a five-year retrospective, comparative cross-sectional study carried out among acutely intoxicated patients admitted to TPCC during the spring months (March through May) of 2016-2020. As the only center serving Gharbia governate of Egypt (1942 km^2^), TPCC covers the high population density of 2,668/km^2^. The center in Tanta also serves neighboring areas lacking such facilities.

### Patients

The total number of cases admitted to TPCC was 2,094 patients. The current study included 1,916 cases admitted to the TPCC during the mentioned times, as 178 patients were excluded due to incomplete medical records. Also, those treated as out-patients were excluded.

### Data Collection

Data collection commenced after obtaining approval from The Research Ethics Committee number 33829 Faculty of Medicine, Tanta University (Tanta, Egypt). Medical records of all cases admitted to the TPCC during the study period were included. The data were handled anonymously to maintain patient confidentiality.

#### Demographic and Clinical Data

Demographic data were collected, including age, sex, occupation, and residence (ie, urban, rural, or from outside the governorate). The patients were classified according to age as follows: neonates aged less than one month; infants aged from one month to less than one year; toddlers aged one to four years; children five to 14 years; adolescents 15-19 years; adults 20-74 years; and older adults aged over 74 years. The manner of intoxication and whether the incident was intentional (ie, accidental, suicidal, or addiction) was documented. The delay time, which is time from ingestion to emergency services, and length of hospital stay (admission-discharge) were reported in hours.

The nature of the toxic substance was determined by referring to the report of the patient or their relatives. Some cases brought the container of toxin so it could be identified directly. The clinical presentation and laboratory investigations were used to confirm the diagnosis. The causative agents were categorized as anticholinesterases, alcohols and drugs of abuse, analgesic antipyretics, phosphides, corrosives and hydrocarbons, Central Nervous System (CNS) depressants, antipsychotics, antidepressants, and cardiovascular drugs and xanthine derivatives. Co-ingestions were also reported.

#### Patient Scoring

The initial condition of the patient on admission was assessed by Glasgow Coma Scale (GCS), and each patient was evaluated on a scale of 15; the patients were classified into severe (GCS 3-8), moderate (GCS 9-13), and mild brain injury (GCS 14-15).^15^ After being stabilized, the poison severity score was calculated for all cases and the patients were categorized according to Persson, et al into minor, mild, moderate, severe, and fatal.^16^ Besides the scoring system, intensive care unit (ICU) admission and discharge type were reported. The patients were classified according to their outcomes into completely cured with favorable outcomes, complicated if long-term sequences were encountered (ie, blindness, stricture formation, or intermediate syndrome), and those with deaths were categorized as unfavorable outcomes.

### Statistical Analysis

Statistical analysis was performed using Statistical Package for the Social Sciences Software Package version 26 (SPSS; IBM Corp.; Armonk, New York USA). The data were presented as means, standard deviation, and percentage. ANOVA test and Bonferroni post hoc test, Chi-square test, Monte Carlo exact test, and Spearman correlation coefficients were used. P values less than .05 were considered statistically significant. Forward linear trend forecasting was used to impose a line of best fit to time series for historical data. Four readings from 2016 to 2019 were used to predict values for 2020 using Microsoft Excel (Microsoft Corp.; Redmond, Washington USA). The percent change from forecasted value was calculated as:

(Actual value for 2020–Forecasted value for 2020)/Actual value for 2020.

The percent change from the previous year was calculated as:

(Actual value for 2020–Previous year value)/Previous year value.

## Results

The majority of patients in this study lived in rural areas, regardless of year, with 66.2% of total admissions coming from rural areas compared to 17.1% from urban areas and 16.7% living outside the governate. During the lockdown period, there were significantly more admissions from urban areas (z = 4.4; P <.001).

In this study, 1,097 females and 819 males were admitted. During the lockdown, 58.8% of the cases were female, consistent with all prior years studied, although the difference in sex distribution by year was not significant. Adults comprised the highest proportion of admissions (42.9%), followed by toddlers (25.1%) and adolescents (21.8%). The lockdown period brought a marked decrease in the number of hospital admissions (243 out of 1,916 cases). Table 1 shows the proportion of the admitted cases according to their ages over the study period. Linear trend forecasting of total admissions during the lockdown showed that the actual number of admissions was less than predicted with a 101.6% decrease and less than the previous year with a 36.1% decrease. Moreover, the current study revealed that 12.3% of total cases admitted during the lockdown were referred to ICU with 50.0% less than the simulated value (Figure 1).

**Table 1.** Distribution and Proportion of the Admitted Cases According to their Ages Over the Period (March through May) of 2016-2020

	N^a^(%^b^)	Age Groups						
		Neonate (<1 Month)	Infants (1 Month – <1 Year)	Toddlers (1 - 4 Years)	Children (5 - 14 Years)	Adolescents (15 - 19 Years)	Adult (20 - 74 Years)	Older Adults (> 74 Years)
**2016**	311	1	3	93	36	70	107	0
	100.0%	0.3%	1.0%	30.0%	11.6%	22.6%	34.5%	0.0%
**2017**	449	0	1	120	38	105	184	0
	100.0%	0.0%	0.2%	26.8%	8.5%	23.4%	41.1%	0.0%
**2018**	533	0	3	145	44	107	233	1
	100.0%	0.0%	0.6%	27.2%	8.3%	20.1%	43.7%	0.2%
**2019**	380	0	3	78	38	75	186	0
	100.0%	0.0%	0.8%	20.5%	10.0%	19.7%	48.9%	0.0%
**2020**	243	0	4	45	22	60	112	0
	100.0%	0.0%	1.6%	18.5%	9.1%	24.7%	46.1%	0.0%
**Total**	**1916**	**1**	**14**	**481**	**178**	**417**	**822**	**1**
	**100.0%**	**0.1%**	**0.7%**	**25.1%**	**9.3%**	**21.8%**	**42.9%**	**0.1%**

a Number of admitted cases.

b The percentage is calculated per row.

**Figure 1A.**
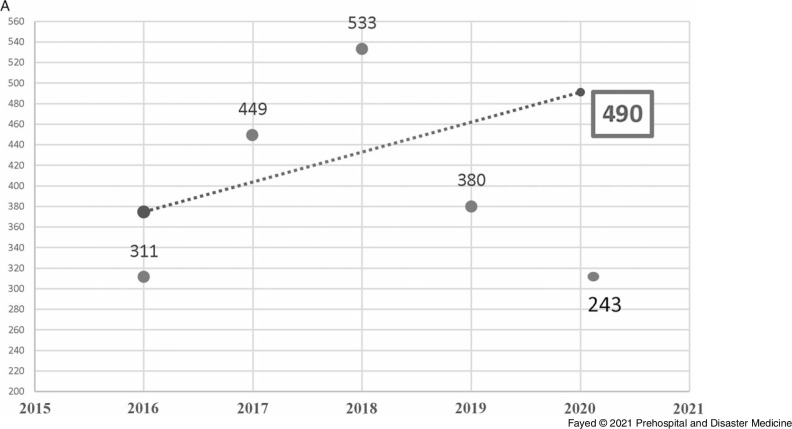
Forecasting the Total Number of Cases Admitted to Tanta Poison Control Center During the Lockdown 2020. Note: The actual number of admissions (243) was less than the predicted number (490) with a 101.6% decrease. The actual number was also less than the previous year (2019) with 36.1% decrease.

**Figure 1B.**
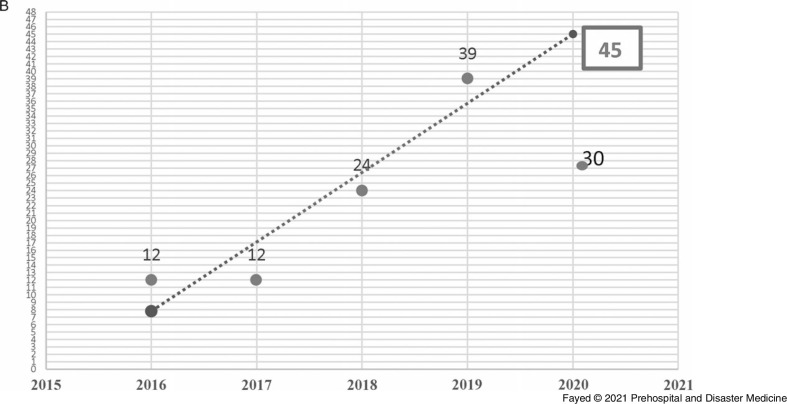
Forecasting the Total Number of Cases Admitted to the ICU, Tanta Poison Center During the Lockdown 2020. Note: The actual number of admissions (30) was less than the predicted number (45) with a 50.0% decrease. The actual number was less than the previous year (2019) with 23.1% decrease. However, when the total number of admissions is considered, the rate of ICU admission is higher during the lockdown period. Abbreviation: ICU, intensive care unit.

**Figure 2.**
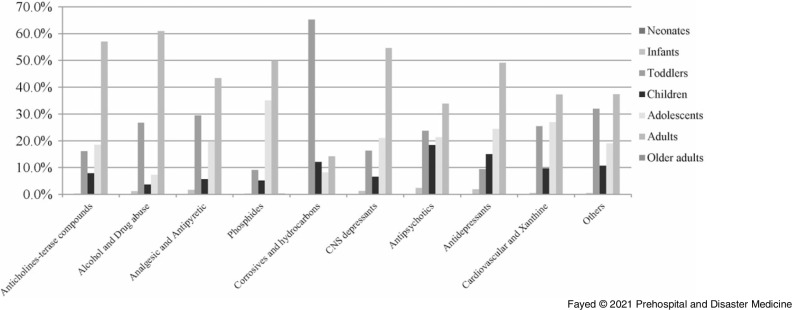
Proportion of the Causative Agent Exposure According to the Age Groups Over the Period (March through May) of 2016-2020. Abbreviation: CNS, Central Nervous System. Anticholinesterase compounds exposure presented as follows: adults (57.0%), adolescents (18.6%), toddlers (16.2%), children (7.9%), and infants (0.3%). Alcohols and drugs of abuse exposure presented as follows: adults (61.0%), toddlers (26.8%), adolescents (7.3%), children (3.7%), and infants (1.2%). Analgesic antipyretic exposure presented as follows: adults (43.4%), toddlers (29.5%), adolescents (19.7%), children (5.7%), and infants (1.7%). Phosphide exposure presented as follows: adults (50.0%), adolescents (35.1%), toddlers (9.1%), children (5.2%), infants (0.3%), and older adults (0.3%). Corrosives and hydrocarbons exposure presented as follows: toddlers (65.3%), adults (14.3%), children (12.2%), and adolescents (8.2%). CNS depressants exposure presented as follows: adults (54.6%), adolescents (21.1%), toddlers (16.4%), children (6.6%), and infants (1.3%). Antipsychotics exposure presented as follows: adults (33.9%), toddlers (23.8%), adolescents (21.4%), children (18.5%), and infants (2.4%). Antidepressants exposure presented as follows: adults (49.1%), adolescents (24.5%), children (15.1%), toddlers (9.4%), and infants (1.9%). Cardiovascular and xanthine derivatives exposure presented as follows: adults (37.3%), adolescents (27.0%), toddlers (25.5%), children (9.7%), and infants (0.5%). Other non-classified toxic exposure presented as follows: adults (37.4%), toddlers (32.0%), adolescents (19.0%), children (10.8%), and infants (0.5%).

Analysis of exposure by age group revealed that adults showed the highest incidence of exposure to all intoxicants, except the corrosives and hydrocarbons, which were significantly more frequent among toddlers (P <.001; Figure 2). Table 2 and Table 3 reveal that adolescents showed significantly more frequent exposure to phosphides (z = 7.1) and significantly less exposure to corrosives (z = -4.9). Children’s exposure to phosphides was low (z = -3.1), while they were significantly more likely to be exposed to antipsychotics (z = 4.3). Regarding infants, antipsychotics exposure was significantly the most frequent (26.7%; z=2.6; P <.001).

**Table 2.** Distribution and Proportion of Causative Agent Exposure According to the Different Age Groups Over the Period (March through May) of 2016–2020

Age Groups N^a^(%^b^^,^^c^)	Causative Agents								
	Anti-Cholinesterase Compounds	Alcohols and Drugs of Abuse	Analgesic and Antipyretic	Phosphides	Corrosives and Hydrocarbons	CNS Depressants	Antipsychotics and Antidepressants		Cardiovascular Drugs & Xanthine Derivatives
**Infants (15)**	1 (6.7%)	1 (6.7%)	2 (13.3%)	1 (6.7%)	0 (0.0%)	2 (13.3%)	4 (26.7%)	1 (6.7%)	1 (6.7%)
**Toddlers (524)**	47 (9.0%)	22 (4.2%)	36 (6.9%)	35 (6.7%)	128 (24.4%)	25 (4.8%)	40 (7.6%)	5 (1.0%)	47 (9.0%)
**Children (191)**	23 (12.0%)	3 (1.6%)	7 (3.7%)	20 (10.5%)	24 (12.6%)	10 (5.2%)	31 (16.2%)	8 (4.2%)	18 (9.4%)
**Adolescents (449)**	54 (12.0%)	6 (1.3%)	24 (5.3%)	135 (30.1%)	16 (3.6%)	32 (7.1%)	36 (8.0%)	13 (2.9%)	50 (11.1%)
**Adults (886)**	166 (18.7%)	50 (6.8%)	53 (6.0%)	192 (21.7%)	28 (3.2%)	83 (9.4%)	57 (6.4%)	26 (2.9%)	69 (7.8%)
**Total 2067** **(100.0%)**	**291 (14.1%)**	**82 (4.0%)**	**122 (5.9%)**	**384 (18.6%)**	**196 (9.5%)**	**152 (7.4%)**	**168 (18.1%)**	**53 (2.6%)**	**185 (9.0%)**

Note: Total number is more than 1916 due to mixed ingestion.

Abbreviation: CNS, Central Nervous System.

a Number of admitted cases.

b Percentage is calculated per row. 100.0% of Neonates (n=1) were exposed to other categories of toxins, 100.0% of older adults (n=1) were exposed to phosphides.

c The percentage adds up to 100.0% when other unclassified drugs is included. Exposure to other unclassified drugs was as follows: 2 (13.3%) of infants, 139 (26.5%) of toddlers, 47 (24.6%) of children, 83 (18.5%) of adolescents, 162 (18.3%) of adults, and 1 (100.0%) of neonates. 434 patients (21.0%) of total admissions were due to exposure to unclassified drug categories.

**Table 3.** Proportion of Causative Agent Exposure According to the Age Groups Over the Period (March through May) of 2016-2020

Causative Agents	Age Groups	
	MC (Monte Carlo Exact Test)	P
**Anticholinesterase Compounds**	30.319	<.001^a^
Toddlers (z score - 3.8)		
Adults (z score 5.3)		
**Alcohols and Drugs of Abuse**	18.116	.006^a^
Adolescents (z score - 3.2)		
Adults (z score 5.4)		
**Phosphides**	108.348	<.001^a^
Toddlers (z score - 8.1)		
Children (z score - 3.1)		
Adolescents (z score 7.1)		
Adults (z score 3.1)		
**Corrosives and Hydrocarbons**	204.478	<.001^a^
Toddlers (z score 13.7)		
Adolescents (z score - 4.9)		
Adults (z score - 8.6)		
**Antipsychotics**	27.258	<.001^a^
Infant (z score 2.6)		
Children (z score 4.3)		
Adults (z score - 2.5)		
**Others**	22.255	.001^a^
Toddlers (z score 3.8)		
Adults (z score - 2.7)		

a P value <.05 (statistically significant).

**Figure 3.**
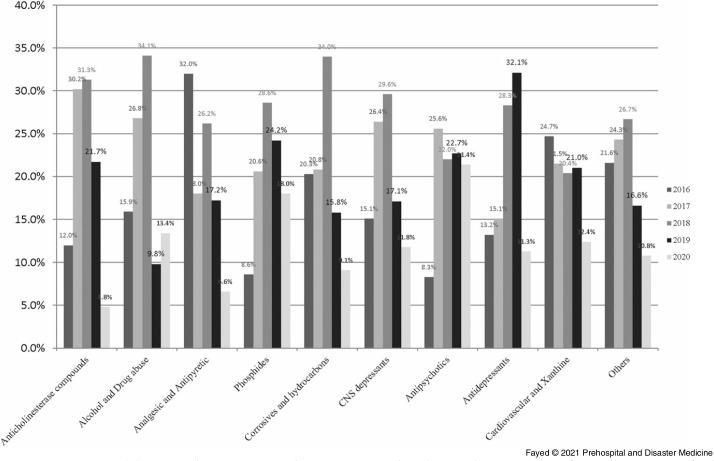
Proportions of Causative Agent Exposure According to the Age Groups Over the Period (March through May) of 2016-2020. Abbreviation: CNS, Central Nervous System. Anticholinesterase compounds exposure presented as follows: 2016 (12.0%), 2017 (30.2%), 2018 (31.3%), 2019 (21.7%), and 2020 (4.8%). Alcohols and drugs of abuse exposure presented as follows: 2016 (15.9%), 2017 (26.8%), 2018 (34.1%), 2019 (9.8%), and 2020 (13.4%). Analgesic antipyretic exposure presented as follows: 2016 (32.0%), 2017 (18.0%), 2018 (26.2%), 2019 (17.2%), and 2020 (6.6%). Phosphide exposure presented as follows: 2016 (8.6%), 2017 (20.6%), 2018 (28.6%), 2019 (24.2%), and 2020 (18.0%). Corrosives and hydrocarbons exposure presented as follows: 2016 (20.3%), 2017 (20.8%), 2018 (34%), 2019 (15.8%), and 2020 (9.1%). CNS depressants exposure presented as follows: 2016 (15.1%), 2017 (26.4%), 2018 (29.6%), 2019 (17.1%), and 2020 (11.8%). Antipsychotics exposure presented as follows: 2016 (8.3%), 2017 (25.6%), 2018 (22.0%), 2019 (22.7%), and 2020 (21.4%). Antidepressants exposure presented as follows: 2016 (13.2%), 2017 (15.1%), 2018 (28.3%), 2019 (32.1%), and 2020 (11.3%). Cardiovascular and xanthine derivatives exposure presented as follows: 2016 (24.7%), 2017 (21.5%), 2018 (20.4%), 2019 (21.0%), and 2020 (12.4%). Other non-classified toxic exposure presented as follows: 2016 (21.6%), 2017 (24.3%), 2018 (26.7%), 2019 (16.6%), and 2020 (10.8%).

The current study revealed that oral exposure was the main route of intoxication (89.6%), followed by inhalation and dermal exposure. Over the study period and regardless of age, phosphide exposure was the most common (18.6%), followed by anticholinesterases (14.1%). The least presented exposure was allocated for alcohols and drugs of abuse (4.0%) and antidepressants (2.6%). Figure 3 shows that 2020 recorded the least proportions of exposure in all drug categories except alcohols and drugs of abuse, phosphides, and antipsychotics.

Table 4 and Table 5 clarify that the lockdown period showed significantly high rates of exposure to phosphides and antipsychotics. On the other hand, exposure to anticholinesterases and analgesic antipyretics was significantly low. Linear trend-forward forecasting of phosphides and anticholinesterase intoxications during the lockdown showed that the actual number of admissions during the lockdown was less than predicted with a large declining percentage, especially among anticholinesterase (percentage of change from the predicted value -550.0% and -77.8% from the previous year), as shown in Figure 4.

**Table 4.** Distributions and Proportions of Causative Agents by Year Over the Period (March through May) of 2016-2020

Year (N^a^)	Causative Agents N_a_(%_b_,_c_)								
	Anti-Cholinesterase Compounds	Alcohols and Drugs of Abuse	Analgesic and Antipyretic	Phosphides	Corrosives and Hydrocarbons	CNS Depressants	Antipsychotics and Antidepressants		Cardiovascular Drugs & Xanthine Derivatives
**2016 (344)**	35 (10.2%)	13 (3.8%)	39 (11.3%)	33 (9.6%)	40 (11.6%)	23 (6.7%)	14 (4.1%)	7 (2.0%)	46 (13.4%)
**2017 (489)**	88 (18.0%)	22 (4.5%)	22 (4.5%)	79 (16.2%)	41 (8.4%)	40 (8.2%)	43 (8.8%)	8 (1.6%)	40 (8.2%)
**2018 (579)**	91 (15.7%)	28 (4.8%)	32 (5.5%)	110 (19.0%)	67 (11.6%)	45 (7.8%)	37 (6.4%)	15 (2.6%)	38 (6.6%)
**2019 (408)**	63 (15.4%)	8 (2.0%)	21 (5.1%)	93 (22.8%)	31 (7.6%)	26 (7.4%)	38 (9.5%)	17 (4.2%)	39 (9.6%)
**2020 (250)**	14 (5.6%)	11 (4.4%)	8 (3.1%)	69 (27.6%)	18 (7.2%)	18 (7.2%)	36 (14.4%)	6 (2.4%)	23 (9.2%)
**Total (2070)**	**291 (14.1%)**	**82 (4.0%)**	**122 (5.9%)**	**384 (18.6%)**	**197 (9.5%)**	**152 (7.3%)**	**168 (8.1%)**	**53 (2.6%)**	**186 (8.9%)**

Note: Total number is more than 1916 due to mixed ingestion.

Abbreviation: CNS, Central Nervous System.

a Number of admitted cases.

b Percent calculated by row.

c The percentage adds up to 100.0% when other unclassified drugs is included. Exposure to unclassified other drugs was as follow: 94 case (27.3%) of admissions in 2016, 106 cases (21.7%) of admissions in 2017, 116 case (20.0%) of admissions in 2018, 72 cases (17.6%) of admissions in 2019, 47 (18.8%) of admissions in 2020. This represents 435 cases (21.0%) of admissions over the study period.

**Table 5.** Distributions of the Causative Agents’ Exposure Over the Period (March through May) of 2016-2020

Causative Agents	Years	
	χ^2^ (Chi Square Test)	P
**Anticholinesterase Compounds**	29.324	<.001^a^
2016 (z = - 2.1)		
2017 (z = 3.0)		
2020 (z = - 4.4)		
**Analgesic and Antipyretic**	25.922	.006 ^a^
2016 (z = 4.9)		
2020 (z = - 2.1)		
**Phosphides**	34.295	<.001^a^
2016 (z = - 4.5)		
2019 (z = 2.4)		
2020 (z = 3.5)		
**Corrosives and Hydrocarbons**	9.949	.041^a^
2018 (z = 2.0)		
**Antipsychotics**	21.494	<.001^a^
2016 (z = - 2.9)		
2020 (z = 3.6)		
**Cardiovascular Drugs & Xanthine Derivatives**	13.687	.008^a^
2016 (z = 3.3)		
2018 (z = - 2.4)		
**Others**	15.124	.004^a^
2016 (z = 3.5)		
2019 (z = - 2.0)		

a P value <.05 (statistically significant).

**Figure 4A.**
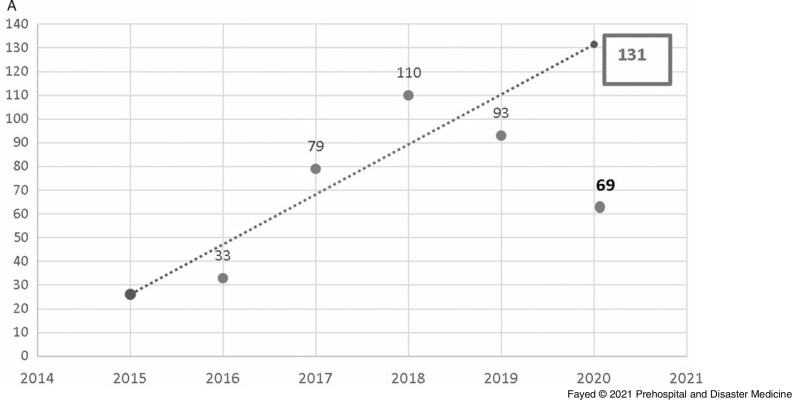
Forecasting the Number of Cases Admitted to Tanta Poison Center due to Phosphide Exposure During the Lockdown 2020. Note: Actual value for 2020: 69 cases, forecasted value for 2020: 131 cases, change from forecasted value: -62, with -89.9% change from forecasted value. Previous year value: 93 cases, change from previous year: -24, with -25.8% change from previous year.

**Figure 4B.**
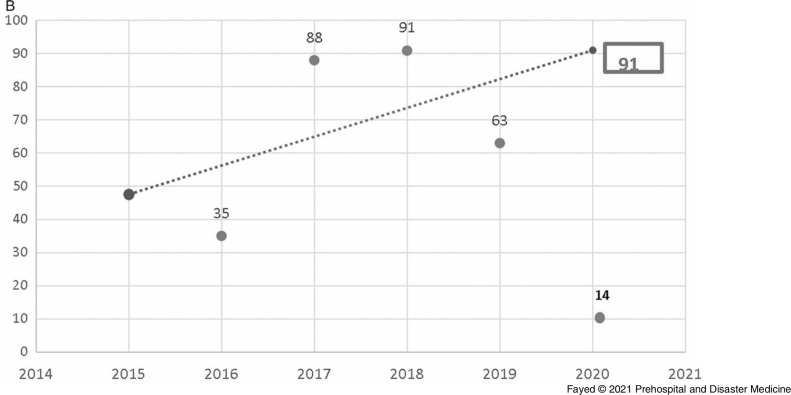
Forecasting the Number of Cases Admitted to Tanta Poison Center due to Anticholinesterase Exposure During the Lockdown 2020. Note: Actual value for 2020: 14 cases, forecasted value for 2020: 91 cases, change from forecasted value: -77, with -550.0% change from forecasted value. Previous year value: 63 cases, change from previous year: -49 with -77.8% change from previous year.

Regarding the drug exposure among females, phosphide exposure was the most frequent (243 cases), followed by cardiovascular drugs and xanthine derivatives (145 cases). Table 6 illustrates that during the lockdown, exposure to anticholinesterases was significantly less frequent than the previous years (P = .02) and exposure to antipsychotics was significantly elevated (z = 3.1; P = .01). Concerning the male’s admission and as shown in Table 7, anticholinesterase exposure was the most frequent (184 cases) followed by phosphides. During the lockdown, there was significantly more phosphides and fewer cases of anticholinesterase exposure among males (P <.001).

**Table 6.** Distribution of Causative Agent Exposure in the Females Admitted to Tanta Poison Center Over the Period (March through May) of 2016-2020

Causative Agents in Females	Years	
	χ^2^ (Chi Square Test)	P
**Anticholinesterase Compounds** **2020 (z = - 2.4)**	11.536	.021^a^
**Analgesic and Antipyretic** **2016 (z = 4.0)**	17.523	.002^a^
**Phosphides** **2016 (z = - 3.9)**	16.723	.002^a^
**Corrosives and Hydrocarbons** **2017 (z = - 2.2)** **2018 (z = 2.1)**	9.621	.047^a^
**Antipsychotics** **2016 (z = - 2.9)** **2020 (z = 3.1)**	18.823	.001^a^
**Cardiovascular Drugs & Xanthine Derivatives** **2016 (z = 3.0)**	10.133	.038^a^
**Others** **2016 (z = 3.6)**	16.811	.002^a^

a P value <.05 (statistically significant).

**Table 7.** Proportions of Causative Agent Exposure in the Males Admitted to Tanta Poison Center Over the Period (March through May) of 2016-2020

Year	Causative Agent in Males N_a_(%_b_,_c_)								
	Anti-Cholinesterase Compounds	Alcohols and Drugs of Abuse	Analgesic and Antipyretic	Phosphides	Corrosives and Hydrocarbons	CNS Depressants	Antipsychotics and Antidepressants		Cardiovascular Drugs & Xanthine Derivatives
**2016**	23	11	12	12	20	8	8	1	9 22.0%
	12.5%	19.0%	30.8%	8.5%	18.5%	14.3%	11.0%	9.1%	
**2017**	56	13	7	24	29	18	17	0	12 29.3%
	30.4%	22.4%	17.9%	17.0%	26.9%	32.1%	23.3%	0.0%	
**2018**	55	19	9	38	34	16	18	5	6 14.6%
	29.9%	32.8%	23.1%	27.0%	31.5%	28.6%	24.7%	45.5%	
**2019**	42	6	9	36	16	8	16	4	10 24.4%
	22.8%	10.3%	23.1%	25.5%	14.8%	14.3%	21.9%	36.4%	
**2020**	8	9	2	31	9	6	14	1	4 9.8%
	4.3%	15.5%	5.1%	22.0%	8.3%	10.7%	19.2%	9.1%	
**Total**	184	58	39	141	108	56	73	11	41 100.0%
	100.0%	100.0%	100.0%	100.0%	100.0%	100.0%	100.0%	100.0%	

Abbreviation: CNS, Central Nervous System.

a Number of admitted cases.

b Percentage is calculated per column.

c The percentage adds up to 100% when other unclassified drugs is included. Exposure of males to other unclassified drugs was distributed as follows: 26 cases (17.7%) in 2016, 34 cases (23.1%) in 2017, 47 cases (32.0%) in 2018, 24 cases (16.3%) in 2019, and 16 cases (10.9%) in 2020. 147 males exposed to other drugs during the study period.

In the current study, co-ingestion was reported in 6.4% of cases. During the lockdown period, 4.9% of cases reported mixed ingestion with no significant differences when compared with other years. Analgesic antipyretics, sedative-hypnotics, antidepressants, and antipsychotics were the most commonly co-ingested xenobiotics.

**Figure 5.**
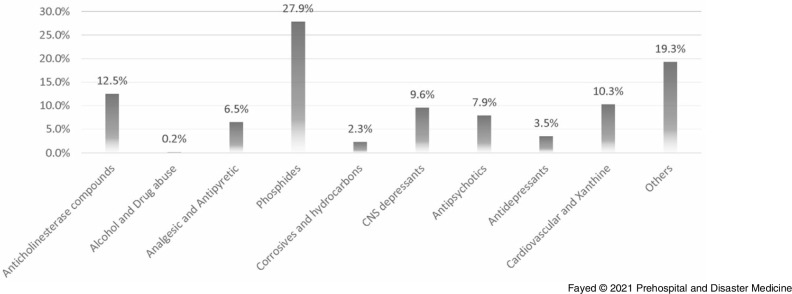
Proportions and Distribution of the of Cases Admitted to Tanta Poison Center due to Suicidal Exposure Over the Period (March through May) of 2016-2020. Abbreviation: CNS, Central Nervous System. Note: Suicidal exposure was attributed to phosphide exposure (27.9%), anticholinesterase compounds exposure (12.5%), cardiovascular and xanthine derivatives (10.3%), CNS depressants (9.6%), antipsychotics (7.9%), analgesic antipyretic (6.5%), antidepressants (3.5%), corrosives and hydrocarbons (2.3%), and alcohols and drug of abuse (0.2%).

The current study revealed that the majority of admitted cases were due to suicidal issues (56.2%). Accidental exposure was second in frequency (39.6%), while admission due to addiction overdose was the least frequent (4.2%), a pattern that remained consistent in all study years (Table 8). The study showed that the majority of suicidal admissions were females (70.1%), while most admissions of males were due to addiction-related overdose (71.6%). Accidental exposure did not show such great variation, as 57.8% were males. The admissions due to suicidal issues during the lockdown period showed similar distribution with more females than males (69.0% females versus 31.0% males). Table 9 shows that corrosives and hydrocarbons constituted the most frequent causes of accidental exposure (21.5%). Figure 5 shows that phosphides were the most involved agents in suicidal exposure (27.9%), followed by anticholinesterase compounds (12.5%).

**Table 8.** Proportions and Distribution of the of Cases Admitted to Tanta Poison Center According to the Manner of Exposure Over the Period (March through May) of 2016-2020

Year	N_a_(%_**b**_)	Manner of Poisoning			Chi-Square Test	P Value
		Accidental	Suicidal	Addiction Overdose		
**2016**	311	145	153	13	**χ**^2^ **26.264**	.009^c^
	100.0%	46.6%	49.2%	4.2%		
**Z Score**		**2.8**	**− 2.7**	—		
**2017**	449	174	253	22		
	100.0%	38.8%	56.3%	4.9%		
**2018**	533	221	285	27		
	100.0%	41.5%	53.5%	5.1%		
**2019**	380	132	240	8		
	100.0%	34.7%	63.2%	2.1%		
**Z Score**		**− 2.2**	**3.1**	**− 2.3**		
**2020**	243	87	145	11		
	100.0%	35.8%	59.7%	4.5%		
**Total**	**1916**	**759**	**1076**	**81**		
	**100.0%**	**39.6%**	**56.2%**	**4.2%**		

Note: The proportion of suicidal admissions during 2019 was significantly higher than in other years (63.2%; z = 3.1; χ^2^ = 26.264; P = .09). The proportion of accidental exposures was significantly higher in 2016 than in other years (46.6%; z = 2.8).

a Number of admitted cases.

b Percentage is calculated per row.

c P value <.05 (statistically significant).

**Table 9.** Proportions and Distribution of the of Cases Admitted to Tanta Poison Center According to the Manner of Exposure Over the Period (March through May) of 2016-2020

Manner of Poisoning (N^a^)	Causative Agents N^a^(%^b^^,^^c^)								
	Anti-Cholinesterase Compounds	Alcohols and Drugs of Abuse	Analgesic and Antipyretic	Phosphides	Corrosives and Hydrocarbons	CNS Depressants	Antipsychotics Antidepressants		Cardiovascular Drugs & Xanthine Derivatives
**Accidental (788)**	141 (17.9%)	0 (0.0%)	44 (5.6%)	49 (6.2%)	169 (21.5%)	34 (4.3%)	73 (9.3%)	11 (1.4%)	63 (8.0%)
**Suicidal (1199)**	150 (12.5%)	2 (0.2%)	78 (6.5%)	335 (27.9%)	28 (2.3%)	115 (9.6%)	95 (7.9%)	42 (3.5%)	123 (10.3%)
**Addiction Overdose (83)**	0 (0.0%)	80 (96.4%)	0 (0.0%)	0 (0.0%)	0 (0.0%)	3 (3.6%)	0 (0.0%)	0 (0.0%)	0 (0.0%)
Total (2070)	291 (14.1%)	82 (4.0%)	122 (5.9%)	384 (18.6%)	197 (9.5%)	152 (7.3%)	168 (8.1%)	53 (2.6%)	186 (9.0%)

Note: Total number is more than 1916 due to mixed ingestion.

Abbreviation: CNS, Central Nervous System.

a Number of admitted cases.

b Percentage is calculated per row.

c The percentage adds up to 100.0% when other unclassified drugs is included. Exposure to unclassified drug categories was as follows: 204 cases (25.9%) of cases admitted due to accidental exposure, 231 cases (19.3%) of cases admitted due to suicidal exposure. Exposure to other unclassified drugs reported in 435 case 21.0% of all admitted cases.

Linear forward forecasting of the manner of poisoning during lockdown is shown in Figure 6. The actual numbers of suicidal, accidental, and addiction overdose exposures were less than the forecasted values. Suicidal admissions during the lockdown period decreased by 110.3% from the forecasted value and by 39.6% from the previous year. Unplanned admissions during the lockdown decreased by 95.4% from the previous years and by 34.1% from 2019. Regarding the admission due to addiction overdose, 11 cases were admitted in comparison to 15 predicted cases and eight cases in 2019.

**Figure 6A.**
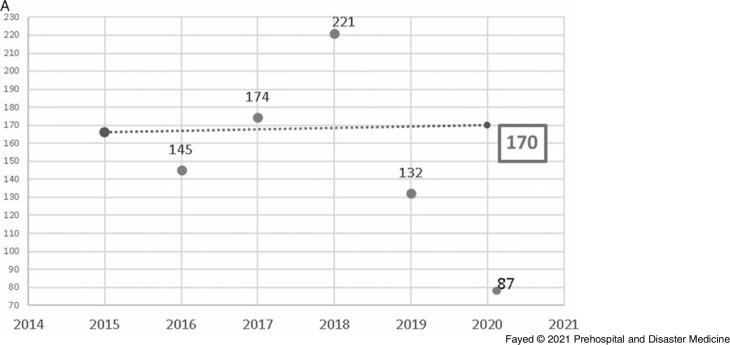
Forecasting the Number of Admissions to Tanta Poison Center due to Accidental Exposure During the Lockdown 2020. Note: Actual value for 2020: 87 cases, forecasted value for 2020: 170 cases, change from forecasted value: -83, with -95.4% change from forecasted value. Previous year value:132 cases, change from previous year: -45, with 34.1% change from previous year.

**Figure 6B.**
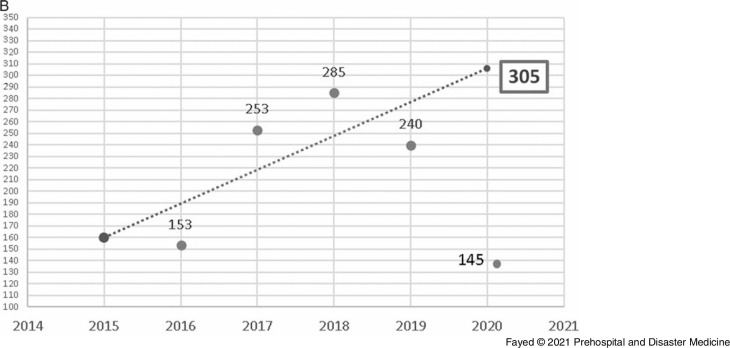
Forecasting the Number of Admissions to Tanta Poison Center due to Suicidal Exposure During the Lockdown 2020. Note: Actual value for 2020: 145 cases, forecasted value for 2020: 305 cases, change from forecasted value: -160, with -110.3% change from forecasted value. Previous year value: 240 cases, change from previous year: -95, with -39.6% change from previous year.

**Figure 6C.**
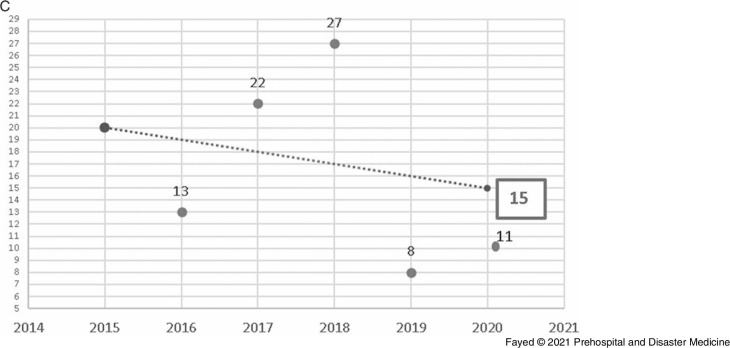
Forecasting the Number of Admissions to Tanta Poison Center due to Addiction Overdose During the Lockdown 2020. Note: Actual value for 2020: 11cases, forecasted value for 2020: 15 cases, change from forecasted value: -3, with -27.3% change from forecasted value. Previous year value: 8 cases, change from previous year: -3, with -37.5 %change from previous year.

Table 10 shows that the lockdown period influenced the delay time obviously as it was significantly increased in comparison to the previous four years (mean: 6.3; P <.001). Bonferroni test revealed that during the lockdown, the delay time to reach the health care facility was significantly prolonged in comparison to previous four years (P <.00). Besides, the current study revealed that the length of hospital stays ranged from 0.1-240 hours. The length of stay varied significantly by year (P <.001). These differences were greatest in 2016, which showed a significantly longer average length of hospital stay than 2018, 2019, and 2020 (P = .002, .009, and .009). Phosphide and analgesics antipyretics intoxicated cases showed the highest length of hospital stay (18.8 [SD = 20.76] and 18.2 [SD = 26.13] hours). Admissions due to antidepressants showed the briefest length of hospital stay (8.9 [SD = 7.14] hours). Linear forward forecasting for the delay time during the lockdown clarifies that the actual value was more than the predicted value by 33.3% and more the previous year by 65.8% (Figure 7A). Furthermore, the cases admitted during the lockdown spent more time in the hospital than the predicted by 17.1%, and slightly less time than the previous year 2019 (12.9) by a difference of 4.6%, as shown in Figure 7B.

**Table 10.** The Delay Time (hours) of the of Cases Admitted to Tanta Poison Center Over the Period (March through May) of 2016–2020

The Time Delay (hours)						The Length of Hospital Stays (in hours)				
Year	N^a^	Mean (SD)	95% CI for Mean	Range	ANOVA	N	Mean (SD)	95% CI for Mean	Range	ANOVA
**2016**	297	3.2 (3.06)	(2.87-3.56)	0.5 – 30	**F** **7.142** **p** <.001^b^	310	17.9 (26.48)	(14.99-20.91)	0.1 – 240.0	**F** **5.890** **p** <.001^b^
**2017**	424	4.2 (7.18)	(3.55-4.92)	0.3 120.0		449	16.3 (21.33)	(14.32-18.28)	0.1 – 216.0	
**2018**	490	3.9 (4.43)	(3.48-4.26)	0.0 – 34.0		531	12.6 (16.06)	(11.21-13.95)	0.1 – 96.0	
**2019**	369	3.8 (3.53)	(3.46-4.18)	0.5 – 24.0		377	12.9 (17.73)	(11.09-14.69)	0.3 – 201.0	
**2020**	234	6.3 (15.27)	4.38-8.31)	0.3 - 168.0		243	12.3 (16.99)	(10.15-14.44)	0.1 – 144.0	
**Total**	1814	4.2 (7.22)	(3.82-4.49)	0.0 – 168.0		1910	14.4 (19.88)	(13.46-15.24)	0.1 – 240.0	

a Number of admitted cases.

b P value <.05 (statistically significant).

**Figure 7A.**
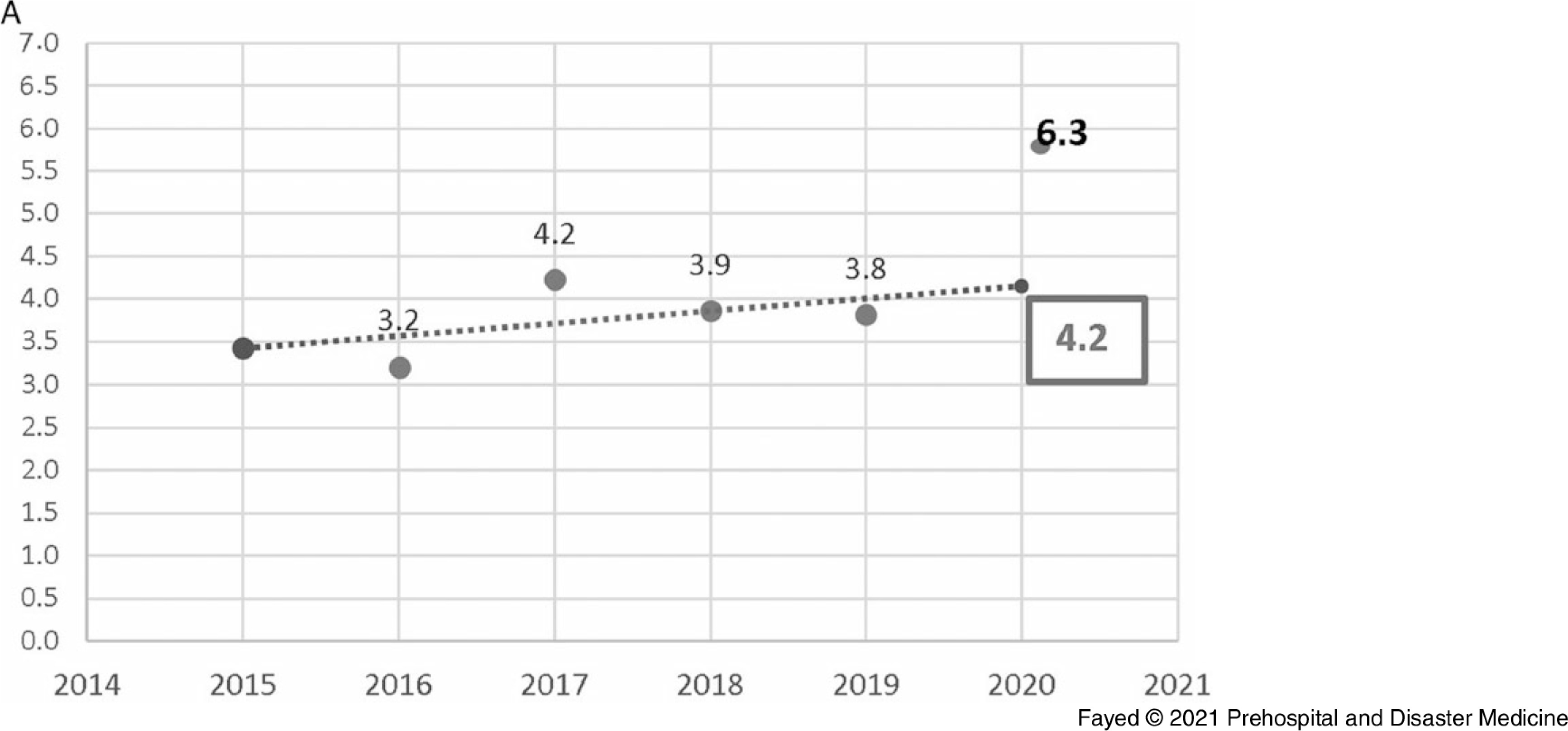
Forecasting the Delay Time (hours) of Cases Admitted to Tanta Poison Center During the Lockdown 2020. Note: Actual value for 2020: 6.3 hours, forecasted value for 2020: 4.2 hours, change from forecasted value: 2.1, with 33.3% change from forecasted value. Previous year value: 3.8 cases, change from previous year: 2.5, with 65.8% change from previous year.

**Figure 7B.**
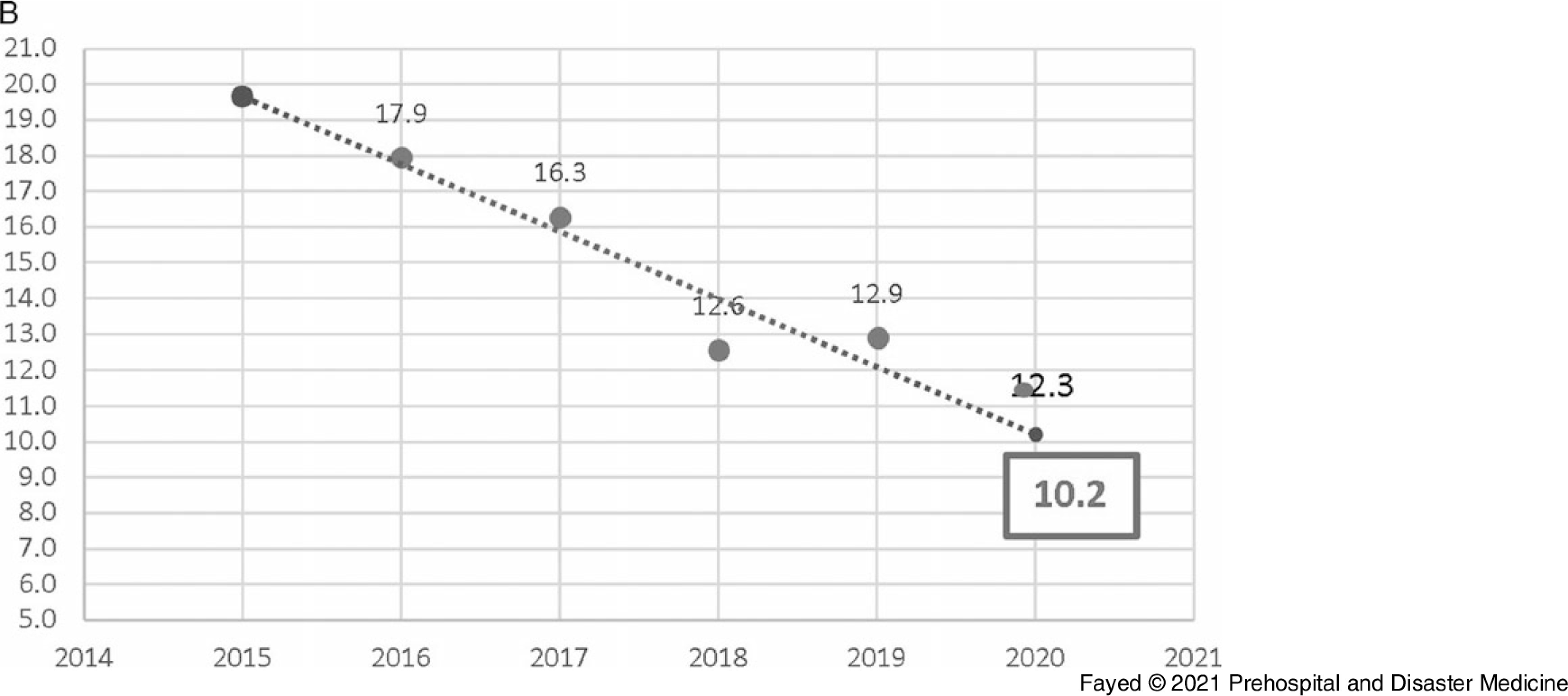
Forecasting the Length of Hospital Stay (hours) of Cases Admitted to Tanta Poison Center During the Lockdown 2020. Note: Actual value for 2020: 12.3 hours, forecasted value for 2020: 10.2 hours, change from forecasted value: 2.1, with 17.1% change from forecasted value. Previous year value: 12.9 hours, change from previous year: -0.6, with -4.6% change from previous year.

Table 11 shows that the majority of admissions were mild cases (93.0%). This distribution was followed in every studied year. The cases admitted during the lockdown period showed significantly lower GCS than those admitted in the previous years (F = 6.300; P <.001). In comparison to the previous years, moderately intoxicated patients’ admissions during the lockdown period were significantly more frequent in comparison to the mild cases, which significantly decreased (z = -6.7). Evaluation by poison severity score yielded similar results; the majority of admitted cases (85.4%) were mild. The least presented categories were the minor cases (3.7%) and the fatal intoxications (0.7%). This pattern was seen in all studied periods, including the lockdown, with no significant differences in between (P = .067).

**Table 11.** Presentation of the Patients Admitted to Tanta Poison Center by Glasgow Coma Scale Over the Period (March through May) of 2016-2020

Year		Mean (SD)	95% CI for Mean	Range	ANOVA	P Value
**2016**		14.7 (1.31)	(14.51-14.80)	3 – 15	**F** **6.300**	<.001^a^
**2017**		14.6 (1.76)	(14.43-14.75)	3 – 15		
**2018**		14.4 **(**2.17)	(14.22-14.59)	3 – 15		
**2019**		14.5 (1.80)	(14.31-14.67)	3 – 15	**Post Hoc. Bonferroni Test**	
**2020**		13.9 (2.27)	(13.64-14.21)	3 – 15	2016 2017 2018 2019	<.001^a^ <.001^a^ .010^a^ .003^a^
**Total**		14.4 (1.91)	(14.35-14.53)	3 – 15		
Year	N	Glasgow Coma Scale Categories			Chi-Square Test	P Value
		Mild	Moderate	Sever		
**2016**	311 (16.3%)	298 (16.7%)	10 (13.7%)	3 (4.9%)	**χ**^2^ **71.074**	<.001^a^
**Z Score**		**2.1**	—	**− 2.4**		
**2017**	449 (23.4%)	428 (24.0%)	10 (13.7%)	11 (18.0%)		
**Z Score**		**2.2**	**2.0**	—		
**2018**	533 (27.8%)	498 (28.0%)	13 (17.8%)	22 (36.1%)		
**2019**	380 (19.8)	357 (20.0%)	9 (12.3%)	14 (23.0%)		
**2020**	243 (12.7%)	201 (11.3%)	31 (42.5%)	11 (18.0%)		
**Z Score**		**- 6.7**	**7.8**	—		
**Total**	**1916 (100%)**	**1782 (100%)**	**73 (100%)**	**61 (100%)**		

a P value <.05 (statistically significant).

Among the admitted patients, 65.6% discharged against medical advice, usually after completion of treatment, and during the observation period, 19.8% discharged due to improvement and 7.4% discharged with further follow-up. Regarding the outcomes of the admitted cases, the current study revealed that among the five years (2016-2020), the recovery rate per 100 population was 92.9, while the death rate was around 3.1. However, approximately four percent of the total admissions showed long-term complications. Table 12 and Table 13 depict that the lockdown period, when compared with previous years, showed significantly lower recovery rates (884.8/1,000 population; z = -3.0) and higher death rates (49.4/1,000 population; z = 2.1). Higher rates of ICU admissions were also associated.

**Table 12.** Distribution and Proportion of the Cases Admitted to Tanta Poison Center According to the Outcome Over the Period (March through May) of 2016-2020

Year	N^a^(%^b^)	Outcomes			Chi-Square Test	P Value
		Favorable	Complicated	Unfavorable (Deaths)		
**2016**	311	296	12	3	**χ**^**2**^ **31.437**	<.001^c^ (Statistically significant)
	100.0%	95.2%	3.8%	1.0%		
**Z Score**		**2.2**	–	**− 3.2**		
**2017**	449	428	5	16		
	100.0%	95.3%	1.1%	3.6%		
**Z Score**		**2.6**	**− 3.5**	–		
**2018**	533	498	21	14		
	100.0%	93.5%	3.9%	2.6%		
**2019**	380	342	23	15		
	100.0%	90.0%	6.1%	3.9%		
**Z Score**		**− 2.6**	**2.1**	–		
**2020**	243	215	16	12		
	100.0%	88.5%	6.6%	4.9%		
**Z Score**		**− 3.0**	**2.1**	**2.0**		
**Total**	**1916**	**1779**	**77**	**60**		
	**100.0%**	**100.0%**	**100.0%**	**100.0%**		

a Number of admitted cases.

b Percentage is calculated by row.

c P value <.05 (statistically significant).

**Table 13.** Recovery, Death, ICU Admission, and Hospital Admission Rates of the Cases Admitted to Tanta Poison Center According to the Outcome Over the Period (March through May) of 2016-2020

	Recovery Rate (per 1,000 population)	Death Rate (per 1,000 population)	ICU Admission Rate (per 1,000 population)	Hospital Admission Rate (per 1,000 population)
**2016**	(296/311) * 1000 = 951.8	(3/311) * 1000 = 9.7	(12/311) * 1000 = 38.6	(311/1916) * 1000 = 162.3
**2017**	(428/449) * 1000 = 953.2	(16/449) * 1000 = 35.6	(12/449) * 1000 = 26.7	(449/1916) * 1000 = 234.3
**2018**	(498/533) * 1000 = 934.3	(14/533) * 1000 = 26.3	(24/533) * 1000 = 45.0	(533/1916) * 1000 = 278.2
**2019**	(342/380) * 1000 = 900.0	(15/380) * 1000 = 39.5	(39/380) * 1000 = 102.6	(380/1916) * 1000 = 198.3
**2020**	(215/243) * 1000 = 884.8	(12/243) * 1000 = 49.4	(30/243) * 1000 = 123.5	(243/1916) * 1000 = 126.8
**Among Past 5 Years**	(1779/1916) * 1000 = 928.5	(60/1916) * 1000 = 31.3	(117/1916) * 1000 = 61.1	(1916/1916) * 1000 = 1000.0

Abbreviation: ICU, intensive care unit.

The current study emphasized that the outcome was influenced by the nature of the intoxicant. Phosphide exposure represented the major cause of mortality (83.7% of total deaths over the study period) and it was directly correlated with unfavorable outcomes (deaths; r_s_ = 0.232; P <.001). Besides, the length of the hospital stay correlated significantly with unfavorable outcome (r_s_ = 0.061; P = .009). Lastly, the increasing age of the patient showed a significant direct correlation with unfavorable outcome (r_s_ = 0.118; P <.001).

## Discussion

This study aimed to characterize the pattern of toxic exposure over the last five years and to explore how the COVID-19 pandemic influenced the pattern of hospital admissions due to toxic exposure reported to the TPCC. This study was carried out during the spring season, which was previously reported to be the peak season for toxic exposures,^17^ although other studies have reported peaks in the winter and fall seasons.^18^

The majority of admitted cases were from rural areas, consistent with previous studies in Egypt and other countries.^19,20^ Lack of health care facilities and private hospitals in rural areas necessitates referral to the cities. Elsewhere, reports indicate a majority of admissions are from urban areas.^21,22^ Differences may also be due to geographical differences between countries. The agricultural nature of the area served by TPCC leads to greater opportunities for pesticide exposure, exacerbated by easy access to the chemicals and inadequate awareness of safety measures. The significant reduction in rural admissions during the lockdown period is best explained by the quarantine measures that restrict transport of those living in remote areas.^23^

In this study, 12.5% of admissions were homemakers, similar to a prior report in India.^13^ In Iran, homemakers are one of the most frequently admitted for intoxication.^24^ A large proportion of homemakers were involved in Turkey, while the proportion of farmers was greater in that study than in the current study. The greater involvement of students with intoxication (57.2%) due to drug poisoning was reported in other studies. In contrast, students were least frequently associated with intoxication admissions in India.^13^

In this study, the total number of hospital admissions during the lockdown was significantly lower than in the four years prior. In Italy, a substantial decrease in pediatric admissions during the lockdown was reported in comparison to the previous year.^12^ COVID-19 pandemic-related decrease in admissions of cardiac patients and for surgical procedures was reported.^25,26^

The current study revealed the predominance of admissions among adults and females, which has been verified elsewhere.^27–30^ Consistent with the frequent involvement of toddlers reported in the current study, the majority of admission in Saudi Arabia was for children under five years of age, although the distribution between male and female patients was nearly equivalent.^31^

Consistent with the current study, the previous literature agreed that oral exposure was the most common route of toxic exposure.^13,29,32,33^ The current study showed that phosphide was the most commonly reported agent, followed by anticholinesterase. A previous study conducted in Egypt revealed that pesticides were the most frequently encountered toxic chemical in children admitted to the poison center, followed by medications and household substances.^33^ Pesticides were also reported as the main cause of morbidity and mortality among patients admitted due to poisoning in developing countries, such as Zimbabwe, Turkey, and Iran.^34,35^ However, exposure to pesticides in developed countries was also encountered.^36^ Alcohol and sedative-hypnotics were the most common causes of admissions for toxic exposure in Australia.^37^

In contrary to the current study, acetaminophen exposure was the most frequently reported agent in Malaysia (35.0%) while pesticides only constituted 6.6% of total admissions.^34^ This pattern was also noted in the United Kingdom^38^ and Saudi Arabia.^31^ Studies in the United States, Turkey, and Oman reported analgesics as the most common cause of drug intoxication.^39^ The conflict between the current study and other studies might be attributed to the predominance of children in the latter cohort.^31^ Country to country variations in prescribing and availability of over-the-counter medications also account for variability in admissions.

However, Al-Barraq and Farahat reported significant exposure to household and hydrocarbons among children in Saudi Arabia.^31^ This finding was consistent with a previous study.^29,33,35,40^ The frequency of children poisonings could be explained by the overactive and curious nature of children, who tend to explore with their mouths. The lower incidence of hydrocarbon intoxications during the lockdown period might be attributed to the increase in supervision and availability of parents and guardians during the pandemic. Pediatric toxic exposure is preventable and can be reduced by enhancing prophylactic measures.^17,41^

In the current study, exposure to CNS-acting drugs (ie, alcohols and drugs of abuse, antidepressants, and sedative-hypnotics) was not uncommon. Previous similar studies described overexposure to CNS-acting drugs as the major cause of intoxications and overdoses. Benzodiazepines were the most frequently involved drugs in Romania, France, and Norway.^22,28,40^ Benzodiazepines and tricyclic antidepressants were the most common cause of hospital admissions in Chile and Japan.^17,42^ The differences between these countries are attributable to differences in the socioeconomic levels.

The significant prevalence of antipsychotic exposure during the lockdown was consistent with another study.^43^ Besides antipsychotics, the current study reported an incremental increase in phosphide exposure during the lockdown, perhaps as a result of the stressful psychological and financial conditions associated with the COVID19 pandemic, such as disturbed sleep rhythms and increased reports of suicidal thoughts.^44^

The co-ingestion of multiple intoxicants was only evident in 6.4% of admitted cases, consistent with previous studies conducted.^31,45,46^ Reports of co-ingestion were higher in other studies.^17,22,28,47^ This disparity might be attributed to the frequent use of alcohol in combination with CNS-acting drugs to obtain a greater intoxicating effect.

The current study reported that suicidal admissions constituted the highest incidence of admitted cases (58.2%), consistent with reports in other countries.^19,21,22,48–52^ Other studies reported accidental poisoning as the most common cause.^29,33,53^ The current study found that females accounted for around 70.0% of suicidal cases. The emotional response of females to stresses might be a justification as males usually use more violent methods for suicide.^54^ The decreased admissions due to suicidal issues during the lockdown period are just a reflection of the decreased total number of cases admitted. Neumann, et al mentioned a decrease in the number of poisoned cases during lockdown, but predicted that with lockdowns and pandemic progression, the suicidal rates might increase due to the resultant depressions.^55^

Suicidal use of pesticides accounts for around one-third of the world’s suicides, with developing countries accounting for a major portion.^56^ Furthermore, pesticide poisoning comes in the second rank of suicides in the Eastern Mediterranean regions.^57^ The current study revealed that the phosphides and organophosphorus compounds were the most commonly used toxins to commit suicides (40.0%). They can be easily obtained in Egypt, sold without restrictions, with lacking proper instructions on the containers. This percent was higher than the estimated proportion by WHO for Africa and the Eastern Mediterranean region, which may be explained by the presence of few published data and deficient records from these areas.^56^ Another factor may be differences in the source of data (urban or rural areas).^58^ In Australia, Turkey, and Iran, most suicidal poisoning was due to drugs rather than pesticides, which might be attributed to the nature of the studied areas.^21,28,30,49^

The current study revealed more delay in presentation to the hospital during the lockdown (mean = 4.2 [SD = 7.22] hours). A Romanian study reported a delay more than six hours attributed to the mild forms of poisoning often being treated first at home.^22^ Studies from England, Spain, and Ethiopia reported shorter delay time with a mean of two to three hours.^59–61^ Patients presenting to the emergency department within two hours of acute poisoning show the least morbidity and mortality.^62^ The delayed presentation during the lockdown could be attributed to preference to avoid hours of a lockdown or difficult access to the transport facility or health care centers during lockdown hours. Studies suggested that delayed presentation more than four hours, and hence delay in initiation of resuscitative measures, could be a possible factor for high mortality, which might explain the increased mortality and unfavorable outcomes during the lockdown period in the present study.^63,64^

On the other hand, the current study showed that the length of hospital stay during the lockdown was longer than the predicted, which represents a higher load on the emergency hospital during pandemics. Despite that, the conveyed length of stay was less than reported elsewhere.^21,22,61,62,65^ This may be attributed to the predominance of mild conscious cases in the current study (93.0%).

## Limitations

Conducting the study in one center is considered a limitation. The excluded cases due to incomplete medical records might affect data analysis; however, data were excluded from the official data base of TPCC.

## Conclusion

COVID-19-associated lockdown greatly reformed the usual intoxication pattern of the cases admitted to emergency room. Higher phosphides and antipsychotics exposure were reported. The lockdown status significantly played a role in delaying time of hospital arrival, which was reflected as lower recovery rates and higher death rates. The current study recommends restricting pesticides handling, raising public awareness regarding hazardous of pesticides, and establishing psychic consultations to reduce suicidal attempts.
